# Impact of prior statin therapy on the outcome of patients with suspected ventilator-associated pneumonia: an observational study

**DOI:** 10.1186/cc13845

**Published:** 2014-04-28

**Authors:** Rémi Bruyere, Clara Vigneron, Sébastien Prin, André Pechinot, Jean-Pierre Quenot, Serge Aho, Laurent Papazian, Pierre-Emmanuel Charles

**Affiliations:** 1 Service de Réanimation Médicale, Hôpital Bocage Central, C.H.U. Dijon, 14 rue Gaffarel, B.P. 77908-21079, Dijon, Cedex, France; 2 Laboratoire de Bactériologie, Plateau Technique de Biologie, C.H.U. Dijon, 2 rue Angélique Ducoudray, B.P. 37013-21070, Dijon, Cedex, France; 3 Service d’Epidémiologie et d’Hygiène Hospitalière, Hôpital Bocage Central, C.H.U. Dijon, 14 rue Gaffarel, B.P. 77908-21079, Dijon, Cedex, France; 4 Service de Réanimation – Détresses respiratoires et infections sévères, Hôpital Nord, Chemin des Bourrely, 13915 Marseille, Cedex 20, France

## Abstract

**Introduction:**

Ventilator-associated pneumonia (VAP) is the most commonly acquired infection in intensive care units (ICU). Its outcome is related, at least in part, to the host’s response. Statins have anti-inflammatory effects and may thus improve the outcome. We aimed to assess the impact of prior statin use in the setting of VAP.

**Methods:**

A six-year cohort study was conducted in a French ICU at a teaching hospital. All of the patients with suspected VAP were included. Baseline characteristics, outcomes, statin exposure, and the description of suspected episodes were collected prospectively. The primary endpoint was 30-day mortality. Patients who were taking statins before admission to the ICU whether or not treatment was continued thereafter (‘previous users’ group) were compared to those without prior statin therapy (‘statin-naive’ group). A survival analysis using a Cox model was conducted in the whole cohort and in the subgroup of prior statin users.

**Results:**

Among the 349 patients included, 93 (26.6%) had taken statins. At baseline, these patients were at higher risk of complications than statin-naive ones (for example, older, more likely to be men and to have underlying diseases, greater simplified acute physiology score II (SAPS II)). There was, however, no difference regarding severity at the time VAP was suspected (sequential organ failure assessment (SOFA): 9.0 (4.0 to 16.0) versus 8.0 (4.0 to 17.0); *P* = 0.11). Nonetheless, 30-day mortality in statin users was not different from that in statin-naive patients (35.5% versus 26.2%, respectively; adjusted hazard ratio (HR) = 1.23 (0.79 to 1.90) 95% confidence interval (CI); *P* = 0.36). In contrast, after limiting analysis to prior statin users and adjusting for potential confounders, those who continued the treatment had better survival than those who did not (HR = 0.47; (0.22 to 0.97) 95% CI; *P* = 0.04).

**Conclusions:**

Statin continuation in prior users could provide protective effects in patients with suspected VAP.

## Introduction

Ventilator-associated pneumonia (VAP) occurs in almost 30% of patients on prolonged mechanical ventilation (MV) [1]. It is therefore the most common nosocomial infection in mechanically ventilated patients and is associated with high morbidity and mortality despite appropriate initial antibiotics [2]. Since MV could promote lung inflammation (that is, biotrauma) leading to ventilator-induced lung injury (VILI), especially if applied on infected lungs, drugs with anti-inflammatory properties are of potential interest in the setting of VAP [3,4]. Moreover, it has been shown that the outcome of patients with VAP was closely related, at least in part, to the degree of both pulmonary and systemic inflammation [5,6].

Statins are lipid-lowering agents that reduce the risk of cardiovascular events by inhibiting 3-hydroxy-3 methyl-glutaryl coenzyme A reductase [7,8]. Immunomodulatory properties have also been described [9]. Although controversial, a number of publications have raised the possibility that these drugs could exert protective anti-inflammatory effects in the context of sepsis, especially pneumonia [10-15]. Interestingly, some experimental studies have demonstrated a lung-protective effect through VILI attenuation [16,17]. However, it is worth noting that the clinical relevance of these findings has not been yet demonstrated [18-20]. The main drawback of the studies published so far, in which the protective effect of statins against sepsis was evaluated, is their retrospective design, which makes it difficult to control for the many clinically relevant and especially ‘protective’ confounders (that is, better health follow-up, greater degree of treatment compliance), since patients with prior statin therapy were always compared with those without. In addition, since sepsis was generally community-acquired in these studies, some significant covariates such as treatment with antibiotics prior to hospital admission may not have been reliably recorded. Moreover, both the type and severity of the infection varied considerably making the case mix somewhat different from one report to another.

In contrast, interesting results were obtained in certain randomized controlled trials (RCTs) that targeted one type of infection and/or one population of patients (for example, intensive care unit (ICU) patients) [20,21]. Thus, Makris *et al.* showed that prior treatment with statins could decrease mortality and ICU length of stay in selected patients with VAP. In contrast, a recent RCT that compared atorvastatin to placebo in patients with suspected VAP has failed to demonstrate any benefit [22].

As a result, statins clinical interest in patients with VAP is uncertain. Moreover, whether statins should be stopped or continued in ICU patients remains an unsolved issue. Since VAP occurs a certain time after ICU admission in therefore closely followed patients, relevant data related to episodes of pneumonia can be considered reliable if collected prospectively, as was the case in our medical ICU [23]. We therefore decided to assess the impact of prior statin therapy on the outcome (that is, ICU all-cause 30-day mortality) of patients with suspected VAP included in our cohort. To this purpose, two distinct sets of analysis were performed. First, we compared the patients with prior statin exposure to those without. Second, we considered only the patients with prior exposure and evaluated mortality according to continuation or discontinuation of the drug in the ICU.

## Materials and methods

### Study population

The database used in this study has already been described elsewhere [23]. Briefly, every patient admitted to our ICU between January, 2006 and January, 2013 was considered if subjected to MV for more than 48 hours. Each patient with suspected VAP according to the physician’s clinical judgment was eligible. However, only those with a ‘modified’ clinical pulmonary infection score (CPIS) of five or greater, provided empirical antibiotics were delivered promptly (that is, day 1), were kept for analysis in the present work, as in the recently published ‘STATIN-VAP’ study, in order to include the patients with a sufficiently high level of suspicion regarding VAP diagnosis [22,24,25].

In accordance with French law, no informed consent was required since all measurements were part of routine management, as confirmed by our local ethics committee (Comité de Protection des Personnes Nord-Est), which gave us its approval for conducting our study.

### Definitions

The patients with suspected VAP were classified into two distinct groups according to prior statin therapy. Thus, the ‘previous users’ group included all of the patients treated with statins prior to ICU admission whether or not they were continued thereafter. The ‘statin-naive’ group included patients without prior statins. For secondary analyses (see below), statin previous users were separated into two groups according to drug continuation until VAP episode (that is, statin continuation or discontinuation in the ICU).

Since tracheal aspirate quantitative cultures were performed, the 10^6^ colony-forming units (CFU)/mL cutoff value was applied for differentiating between positive and negative results.

Bacteria were considered multidrug resistant (MDR) in the following cases according to recent recommendations: (i) *Pseudomonas aeruginosa* resistant to imipenem and/or antipseudomonal penicillins and/or one aminoside and/or ciprofloxacine; (ii) *Enterobacteriacae* if resistant to third-generation cephalosporins and/or fluoroquinolone and/or an aminoside; (iii) *Staphylococcus aureus* if resistant to oxacillin [26]. Patients with negative tracheal aspirate cultures were considered free of MDR bacteria.

Immunosuppression was defined as neutropenia (polymorphonuclear cells counts less than 1500/mm^3^), any immunosuppressive treatment prior to ICU admission including steroids if given for more than one month.

### Data collection

Using a recording form, ‘modified’ CPIS value, demographic data and usually reported risk factors for MDR bacteria were prospectively recorded (that is, time between VAP suspicion and ICU admission, previous hospitalization, exposure to antibiotics defined as the administration of at least one two-day course of antibiotics within the past 30 days, residence in a nursing home, underlying chronic obstructive pulmonary disease). The clinical course of VAP was also assessed through day-1 and day-3 sequential organ failure assessment (SOFA) scores, the duration of mechanical ventilation and the number of ventilator-free days. The primary outcome was 30-day all-cause mortality.

Prior statin therapy was assessed through retrospectively collected data after the prospective cohort had been established. All the available records (both in and out of hospital) were used to determine whether patients had received statins before and after ICU admission. They were considered as previous users if they used to take statins prior to ICU admission regardless of how long they did.

In addition, procalcitonin (PCT) measurement was usually performed in every patient with suspected sepsis as a reliable tool to improve diagnosis and antimicrobial management [27]. Tracheal aspirate samples were taken in every patient within a 24-hour period following the clinical suspicion. The results of the bacterial cultures were used to calculate the ‘day-3 CPIS’ since one point was added to the value obtained at day 1 if at least 10^6^ CFU /mL were recovered. One point was then added if the direct examination showed the same germ.

### VAP management

The guidelines for the antibiotic therapy management were based on the knowledge of local susceptibility patterns of the most frequently isolated bacteria, as well on the clinical judgment of the attending physician. The first-line treatment (that is, the one delivered within the first 24 hours following the clinical suspicion of VAP) was considered appropriate if the isolated pathogen(s) was (were) susceptible to at least one drug administered at the onset of sepsis according to the corresponding susceptibility testing report. When no antibiotic was given within the first 24 hours of management, the treatment was considered inappropriate regardless of the subsequently isolated pathogen.

### Statistical analysis

Values are expressed as median (range) unless otherwise stated.

#### Comparative analysis

First, patients with VAP were compared according to prior statin use as defined above. Continuous variables were compared using the Mann-Whitney *U* test, and categorical variables were compared using the chi-squared test.

#### Survival analysis

Then, 30-day survival analyses were conducted.

First, the whole cohort was considered. The survival of patients regarding statin therapy was analyzed through the construction of the corresponding Kaplan-Meier curves compared by the log-rank test. Then, in addition to statin exposure (that is, ‘previous users’ vs. ‘statin- naive’), every variable associated with 30-day death in the ICU according to the univariate analysis was entered into a multivariate Cox model if the *P* value was less than 0.20.

In the second set of analyses restricted to prior statin users, 30-day survival was evaluated by univariate analysis as described above. Again, the corresponding Kaplan-Meier curve was constructed and a multivariate analysis based on a Cox model was performed in an attempt to withdraw potential confounders. In addition to statin use after admission to the ICU (that is, ‘statin continued’ vs. ‘statin discontinued’), covariates were selected according to the results of the univariate analysis. Only those associated with death with a *P* value less than 0.20 were included into the model.

For all Cox model analyses, scaled Schoenfeld residuals (graphical inspection and formal testing for a nonzero slope in a regression of the residuals on functions of time) were used to check the Cox model, for each variable.

The functional form of continuous variables (V1, V2…) was checked with martingale residual analysis and by means of fractional polynomials.

No *a priori* interactions were clinically indicated or tested.

The goodness-of-fit was assessed by the Cox-Snell residuals, bias-corrected Akaike’s information criterion.

All tests were two-tailed. A *P* value of <0.05 was considered statistically significant. STATA software was used for all analyses (StataCorp, College Station, TX, USA).

## Results

### Patients’ characteristics

**Figure 1:**
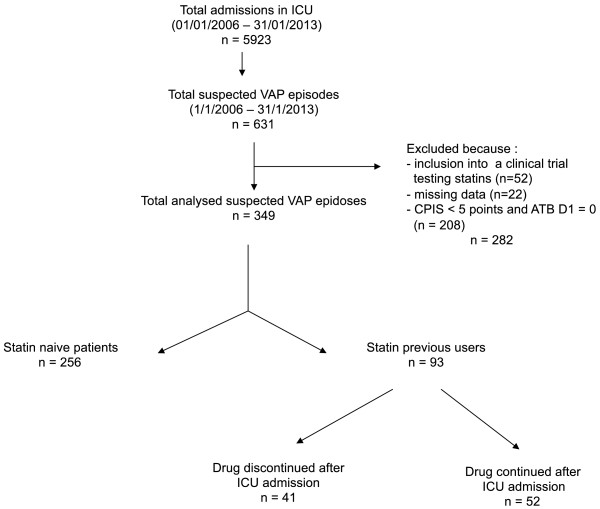
**Flow chart of selection of study patients.** ATB, antibiotics; CPIS, clinical pulmonary infection score; ICU, intensive care unit; VAP, ventilator-associated pneumonia.

Between 1 January 2006 and 31 January 2013, 631 episodes of suspected VAP were recorded. Seventy-four episodes were excluded because of missing data (n = 22), inclusion in a RCT comparing atorvastatin to placebo for the management of VAP (n = 52), CPIS less than five or the absence of antibiotics delivered on day 1 (n = 208). Among the 349 remaining cases, 93 (26.6%) had been treated with statins, while 256 had not (73.4%) (Figure 1). Among the former patients (that is, ‘previous users’), statins were continued until VAP in 52 (55.9%) for a duration of 9.2 (2.0 to 71.0) days.

The main baseline characteristics of the included patients are presented in Table 1. It is worth noting that statin previous users were sicker than statin-naive patients at ICU admission. Actually, the statin patients were significantly older, had higher SAPS II scores on admission, and were more likely to harbor underlying diseases. In contrast, main admission diagnosis was similar.

**Table 1:** Patients with suspected VAP

	**Overall**	**Statin previous users**	**Statin-naive patients**	***P***
	**(n = 349)**	**(n = 93)**	**(n = 256)**	
Age (years)	64.0 (17.0-94.0)	68.0 (27.0-90.0)	63.0 (17.0-94.0)	<0.01
SAPS II	50.0 (6.0-109.0)	54.0 (18.0-109.0)	49.0 (6.0-99.0))	0.06
Gender, male (n (%))	254 (72.8)	80 (86.0)	174 (68.0)	<0.01
Hospitalization prior to ICU admission (n (%))	347 (62.3)	89 (67.9)	258 (60.6)	0.14
Underlying disease(s)				
COPD (n (%))	99 (17.7)	33 (25.1)	66 (15.5)	0.01
Chronic renal failure (n (%))	23 (6.6)	10 (10.9)	13 (5.1)	0.05
Chronic cardiac disease (n (%))	138 (39.6)	65 (70.6)	73 (28.5)	<0.01
Diabetes mellitus (n (%))	67 (19.2)	29 (31.5)	38 (14.8)	<0.01
Cirrhosis (n (%))	17 (4.9)	2 (2.2)	15 (5.9)	0.16
Immunosuppression (excepting steroids) (n (%))	17 (4.9)	4 (4.3)	13 (5.1)	0.78
Steroids (n (%))	20 (5.8)	8 (8.7)	12 (4.7)	0.16
Cancer (n (%))	31 (8.9)	10 (10.9)	21 (8.2)	0.44
Nursing-home resident (n (%))	16 (4.6)	2 (2.1)	14 (5.5)	0.19
Main admission diagnosis				0.20
Respiratory distress (n (%))	133 (38.1)	33 (35.5)	100 (39.1)	0.54
Extrapulmonary sepsis (n (%))	107 (30.6)	33 (35.5)	74 (69.2)	0.24
Neurologic failure (n (%))	50 (14.3)	10 (10.7)	40 (15.6)	0.24
Abdominal surgery (n (%))	17 (4.9)	2 (2.1)	15 (5.8)	0.15
Miscellaneous (n (%))	42 (12.0)	15 (16.1)	27 (10.5)	0.16

Baseline characteristics according to statin exposure. VAP, ventilator-associated pneumonia; SAPS II, simplified acute physiology score II; ICU, intensive care unit; COPD, chronic obstructive pulmonary disease; MV, mechanical ventilation.

### VAP episodes description

Among the 349 episodes of VAP analyzed, a positive bacterial culture was obtained in around 80% of the cases in each group. For the whole population, *Enterobacteriaceae* were the most frequently isolated pathogen (29.9%). This proportion reached 33.5% in the patients with statins.

Overall, MDR germs, as defined above, were isolated in 33.1% of patients with statins and 28.6% of those without (*P* = 0.33).

The first-line antibiotic therapy was appropriate in most of the cases (76.9% and 76.2% in the ‘statin previous users’ and in the ‘statin-naive’ groups, respectively; *P* = 0.89).

Regarding the severity of VAP episodes, the occurrence of septic shock on day 1 was similar in the two groups (38.0% versus 32.0%, *P* = 0.29), as were the CPIS values and the SOFA scores on day 1 and day 3 (Table 2). Similarly, the same proportions of patients in both groups were given steroids (45.1% vs. 45.0%, respectively; *P* = 0.99).

**Table 2:** Suspected VAP episode description according to statin exposure and outcome

	**Overall**	**Statin previous users**	**Statin-naive patients**	***P***
	**(n = 349)**	**(n = 93)**	**(n = 256)**	
Time elapsed between ICU admission and VAP (days)	10.0 (2.0-157.0)	10.0 (2.0-72.0)	10.0 (2.0-157.0)	0.62
Time elapsed between MV onset and VAP (days)	10.0 (2.0-156.0)	10.0 (2.0-69.0)	10.0 (2.0-156.0)	0.48
Early VAP (n (%))	108 (30.9)	27 (29.0)	81 (31.6)	0.64
Septic shock (n (%))	117 (33.6)	35 (38.0)	82 (32.0)	0.29
Appropriate antibiotic therapy (n (%))	262 (76.4)	70 (76.9)	192 (76.2)	0.89
Concurrent therapy during VAP period				
Steroids (n (%))	153 (45.0)	41 (45.1)	112 (45.0)	0.99
RRT (n (%))	83 (24.4)	29 (31.9)	54 (21.7)	0.05
CPIS D1	6.0 (2.0-11.0)	5.0 (2.0-9.0)	6.0 (2.0-11.0)	0.32
CPIS D3	7.0 (5.0-13.0)	7.0 (5.0-11.0)	7.0 (5.0-13.0)	0.25
SOFA D1	8.0 (4.0-17.01)	9.0 (4.0-16.0)	8.0 (4.0-17.0)	0.11
SOFA D3	8.0 (4.0-18.0)	8.0 (4.0-15.0)	8.0 (4.0-18.0)	0.63
PCT D-1	1.2 (0.1-172.0)	1.1 (0.1-55.9)	1.4 (0.1-172.0)	0.42
PCT D1	1.2 (0.1-91.4)	1.3 (0.1-30.0)	1.2 (0.1-91.4)	0.41
PCT D2	1.5 (0.1-162.0)	1.4 (0.1-64.3)	1.6 (0.1-162.0)	0.28
PCT D3	1.3 (0.1-345.2)	1.5 (0.1-56.2)	1.2 (0.1-345.2)	0.71
PCT D4	1.2 (0.1-199.2)	1.0 (0.1-35.3)	1.2 (0.1-199.2)	0.61
Ventilator-free days	4.0 (0.0-112.0)	2.0 (0.0-30.0)	4.0 (0.0-112.0)	<0.01
Length of ICU stay (days)	26.0 (4.0-204.0)	25.0 (4.0-147.0)	26.0 (5.0-204.0)	0.80
Duration of MV (days)	20.0 (2.0-176.0)	20.0 (2.0-121.0)	19.0 (2.0-176.0)	0.42
30-day mortality (n (%))	100 (28.6)	33 (35.5)	67 (26.2)	0.09

VAP, ventilator-associated pneumonia; ICU, intensive care unit; MV, mechanical ventilation; RRT, renal replacement therapy; CPIS, clinical pulmonary infection score; D, day; SOFA, sequential organ failure assessment; PCT, procalcitonin.

No significant difference was found regarding PCT measurements.

### Survival analysis

**Figure 2:**
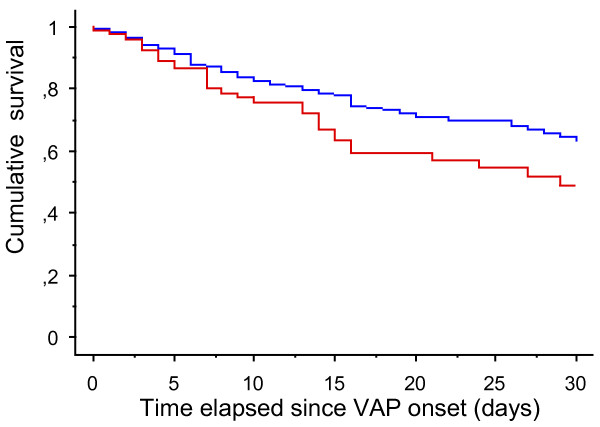
**Survival analysis of patients with suspected VAP according to the prior use of statin.** Red-line: ‘statin previous users’; blue-line: ‘statin-naive patients’ (log-rank test: *P* = 0.04). VAP, ventilator-associated pneumonia.

The overall mortality rate for the whole cohort reached 28.6%. Prior statin therapy did not modify the outcome (Tables 2 and 3) (Figure 2).

**Table 3:** Baseline characteristics and suspected VAP episode description of the study patients according to the 30-day mortality

	**Survivors**	**Nonsurvivors**	***P***
	**(n = 249)**	**(n = 100)**	
Age (years)	62.0 (17.0-94.0)	68.5 (32.0-90.0)	<0.01
SAPS II (points)	47.0 (6.0-109.0)	55.0 (15.0-99.0)	<0.01
Gender, male (n (%))	182 (73.1)	72 (72.0)	0.83
Underlying disease(s) (n (%))			
Chronic renal failure	12 (4.8)	11 (11.0)	0.04
Cardiac chronic disease	84 (33.9)	54 (54.0)	<0.01
Diabetes mellitus	45 (18.1)	22 (22.0)	0.41
COPD	45 (18.1)	21 (21.0)	0.53
Cirrhosis	6 (2.4)	11 (11.0)	<0.01
Immunosuppression	9 (3.6)	8 (8.0)	0.09
Cancer	25 (10.1)	6 (6.0)	0.21
Nursing-home resident (n (%))	16 (6.4)	0 (0.0)	<0.01
Main admission diagnosis			0.02
Respiratory distress (n (%))	101 (40.6)	32 (32.0)	0.13
Extrapulmonary sepsis (n (%))	64 (25.7)	43 (43.0)	<0.01
Neurologic failure (n (%))	40 (16.1)	10 (10.0)	0.14
Abdominal surgery (n (%))	14 (5.6)	3 (3.0)	0.30
Miscellaneous (n (%))	30 (12.0)	12 (12.0)	0.99
CPIS day 1	6.0 (2.0-11.0)	5.0 (3.0-9.0)	0.06
CPIS day 3	7.0 (5.0-13.0)	7.0 (5.0-11.0)	0.20
SOFA day 1	7.0 (4.0-17.0)	10.0 (4.0-16.0)	<0.01
SOFA day 3	7.0 (4.0-16.0)	9.0 (4.0-18.0)	<0.01
Length of ICU stay until VAP (days)	10.0 (2.0-157.0)	11.0 (2.0-72.0)	0.48
Duration of MV until VAP (days)	10.0 (2.0-156.0)	10.0 (2.0-70.0)	0.37
Septic shock (VAP day 1) (n (%))	71 (28.6)	46 (46.0)	<0.01
Late-onset VAP (≥5 days after MV onset) (n (%))	166 (66.7)	75 (75.0)	0.13
MDR bacteria (n (%))	76 (30.8)	41 (41.0)	0.05
Appropriate antibiotic therapy within the first 24 hours of VAP (n (%))	193 (78.4)	69 (69.0)	0.16
Steroids during VAP period (n (%))	89 (36.8)	64 (64.0)	<0.01
Statin exposure prior to ICU admission (n (%))	60 (24.1)	33 (33.0)	0.09
Statin continuation after ICU admission (n (%))	37 (14.8)	15 (15.0)	0.97

VAP, ventilator-associated pneumonia; SAPS II, simplified acute physiology score II; COPD, chronic obstructive pulmonary disease; CPIS, clinical pulmonary infection score; SOFA, sequential organ failure assessment; ICU, intensive care unit; MV, mechanical ventilation; MDR, multidrug resistant.

To eliminate potential confounders, a time-dependent multivariate analysis based on a Cox model was conducted. Statin therapy was found not to have any protective effect (Table 4). Conversely, as expected, immunosuppression, age, cirrhosis, high SAPS II, SOFA day-1 values and the use of steroids during the VAP episode were independently associated with decreased survival in the ICU.

**Table 4:** Independent predictors of 30-day mortality in patients with suspected VAP

	**Hazard ratio**	**95% CI**	***P***
Steroids during VAP	1.93	1.25-2.98	<0.01
Cirrhosis	3.72	1.83-4.31	<0.01
Age	1.02	1.01-1.04	<0.01
SAPS II	1.02	1.01-1.04	<0.01
SOFA day 1	1.15	1.07-1.23	<0.01
Statin prior exposure	1.23	0.79-1.90	0.36

VAP, ventilator-associated pneumonia; CI, confidence interval; SAPS II, simplified acute physiology score II; SOFA, sequential organ failure assessment.

We conducted additional survival analyses within the subset of patients in whom statins previous users (n = 93). The patients in whom statins were continued once admitted to the ICU (n = 52) were distinguished from the remaining ones (n = 41).

First, we compared those patients with respect to their baseline characteristics and main admission diagnosis as well in an attempt to identify potential factors likely to have influence on the decision to stop statin therapy in the ICU (Table 5). No difference was found between groups, except hospitalization prior to ICU admission that was found to be more frequent in the patients in whom statins were discontinued.

**Table 5:** Patients with suspected VAP and previous exposure to statins

	**Statin continuation**	**Statin discontinuation**	***P***
	**(n = 52)**	**(n = 41)**	
Age (years)	67.5 (27.0-85.0)	68.0 (46.0-90.0)	0.99
SAPS II	51.0 (18.0-109.0)	55.0 (24.0-91.0)	0.56
Gender, male (n (%))	44 (84.6)	36 (87.8)	0.66
Hospitalization prior to ICU admission (n (%))	31 (59.6)	33 (80.5)	0.03
Underlying disease(s)			
COPD (n (%))	10 (19.2)	14 (34.1)	0.10
Chronic renal failure (n (%))	6 (11.8)	4 (9.7)	0.76
Chronic cardiac disease (n (%))	37 (72.5)	28 (68.3)	0.66
Diabetes mellitus (n (%))	20 (39.2)	9 (21.9)	0.07
Cirrhosis (n (%))	0 (0.0)	2 (4.9)	0.11
Immunosuppression (excepting steroids) (n (%))	2 (4.9)	2 (3.9)	0.82
Steroids (n (%))	5 (9.8)	3 (7.3)	0.67
Cancer (n (%))	6 (11.8)	4 (9.8)	0.76
Nursing-home resident (n (%))	1 (1.9)	1 (2.4)	0.86
Main admission diagnosis			0.20
Respiratory distress (n (%))	22 (42.3)	11 (26.8)	0.12
Extrapulmonary sepsis (n (%))	15 (28.8)	18 (43.9)	0.13
Neurologic failure (n (%))	4 (7.7)	6 (14.6)	0.28
Abdominal surgery (n (%))	0 (0.0)	2 (4.9)	0.11
Miscellaneous (n (%))	11 (21.1)	4 (9.8)	0.14
Renal failure on admission (Yes (%))	29 (55.8)	21 (51.2)	0.66
Nasogastric tube (Yes (%))	52 (100)	39 (95.1)	0.11

Baseline characteristics according to statin continuation. VAP, ventilator-associated pneumonia; SAPS II, simplified acute physiology score II; ICU, intensive care unit; COPD, chronic obstructive pulmonary disease.

**Figure 3:**
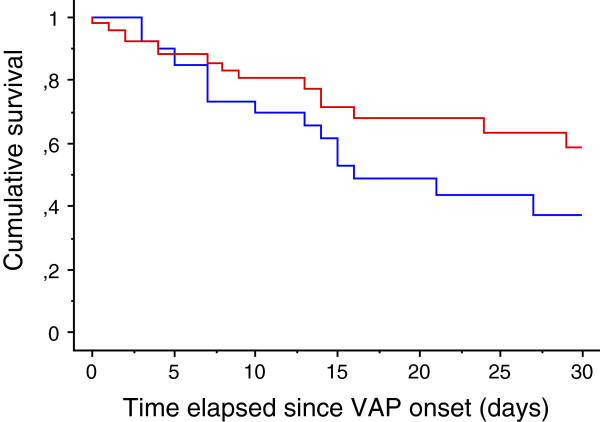
**Survival analysis of patients with suspected VAP in the statin prior users subset according to drug continuation.** Red-line: ‘statin continuation’; blue-line: ‘statin discontinuation’ (log-rank test: *P* = 0.13). VAP, ventilator-associated pneumonia.

The mortality rate tended to be lower in patients who continued statin therapy than in the others (28.8% vs. 43.9%; *P* = 0.13) (Table 6). Moreover, after adjusting for age and SOFA day-1 values, statin continuation in prior users was found to be significantly associated with an improved outcome (adjusted hazard ratio (HR) = 0.47; (0.22 to 0.97) 95% confidence interval (CI); *P* = 0.04) (Table 7) (Figure 3). Similar findings were obtained when the analysis was restricted to the only patients with positive quantitative cultures (n = 73), that is those with confirmed VAP (Table 8).

**Table 6:** Baseline characteristics and VAP episode description in the statin previous users according to the 30-day mortality

	**Survivors**	**Nonsurvivors**	***P***
	**(n = 60)**	**(n = 33)**	
Age (years)	62.5 (27.0-81.0)	74.0 (50.0-90.0)	<0.01
SAPS II (points)	48.0 (18.0-109.0)	57.0 (30.0-86.0)	0.01
Gender, male (n (%))	50 (83.3)	30 (90.9)	0.31
Hospitalization prior to ICU admission (n (%))	41 (68.3)	23 (69.7)	0.89
Underlying disease(s) (n (%))			
Chronic renal failure	5 (8.5)	5 (15.1)	0.32
Cardiac chronic disease	38 (64.4)	27 (81.8)	0.08
Diabetes mellitus	19 (32.2)	10 (30.3)	0.99
COPD	15 (25.0)	9 (27.3)	0.81
Cirrhosis	1 (1.7)	1 (3.3)	0.67
Immunosuppression	4 (6.8)	0 (0.0)	0.13
Cancer	7 (11.9)	3 (9.1)	0.68
Nursing-home resident (n (%))	2 (3.3)	0 (0.0)	0.29
Main admission diagnosis			
Respiratory distress (n (%))	21 (35.0)	12 (36.3)	0.89
Extrapulmonary sepsis (n (%))	19 (31.7)	14 (42.4)	0.30
Neurologic failure (n (%))	6 (10.0)	4 (12.1)	0.75
Abdominal surgery (n (%))	2 (3.3)	0 (0.0)	0.29
Miscellaneous (n (%))	12 (20.0)	3 (9.1)	0.17
Nasogastric tube (Yes (%))	58 (96.7)	33 (100)	0.30
CPIS day-1	5.0 (2.0-9.0)	5.0 (3.0-8.0)	0.38
CPIS day-3	7.0 (5.0-11.0)	7.0 (5.0-10.0)	0.30
SOFA day-1	7.0 (4.0-15.0)	10.0 (5.0-16.0)	<0.01
SOFA day-3	7.0 (4.0-14.0)	8.0 (4.0-15.0)	0.08
Renal failure on admission (Yes (%))	30 (50.0)	20 (60.6)	0.33
Serum creatinine (VAP day-1)	90.0 (27.0-404.0)	137.0 (34.0-321.0)	0.02
Serum creatinine (VAP day-3)	84.0 (14.0-332.0)	111.0 (27.0-329.0)	0.20
Length of ICU stay until VAP (days)	10.0 (2.0-60.0)	10.0 (2.0-72.0)	0.90
Duration of MV until VAP (days)	12.0 (2.0-52.0)	7.0 (2.0-69.0)	0.44
Septic shock (VAP day-1) (n (%))	18 (30.5)	17 (51.5)	0.05
Late-onset VAP (≥5 days after MV onset) (n (%))	41 (68.3)	25 (75.8)	0.45
MDR bacteria (n (%))	19 (32.2)	14 (42.4)	0.33
Appropriate antibiotic therapy within the first 24-hour of VAP (n (%))	49 (83.0)	21 (65.6)	0.06
Steroids therapy during VAP period (n (%))	23 (39.6)	18 (54.5)	0.17
Statin continuation after ICU admission (n (%))	37 (61.7)	15 (45.4)	0.13

VAP, ventilator-associated pneumonia; SAPS II, simplified acute physiology score II; ICU, intensive care unit; COPD, chronic obstructive pulmonary disease; CPIS, clinical pulmonary infection score; SOFA, sequential organ failure assessment; MV, mechanical ventilation; MDR, multidrug resistant.

**Table 7:** Independent predictors of 30-day death in the statin previous users subset of patients with clinically suspected VAP

	**Adjusted hazard ratio**	**95% CI**	***P***
Age	1.06	1.02-1.11	<0.01
SOFA day-1	1.16	1.03-1.32	0.02
Statin continuation after ICU admission	0.47	0.22-0.97	0.04

VAP, ventilator-associated pneumonia; CI, confidence interval; SOFA, sequential organ failure assessment; ICU, intensive care unit.

**Table 8:** Independent predictors of 30-day death in the statin previous users subset of patients with clinically suspected VAP and tracheal aspirate positive culture

	**Adjusted hazard ratio**	**95% CI**	***P***
Age	1.10	1.04-1.20	<0.01
SOFA day-1 value	1.24	1.06-1.46	<0.01
Statin continuation after ICU admission	0.30	0.11-0.81	0.02

VAP, ventilator-associated pneumonia; CI, confidence interval; SOFA, sequential organ failure assessment; ICU, intensive care unit.

## Discussion

In addition to their lipid-lowering properties, statins have pleiotropic effects, which are likely to improve the outcome of patients with coronary heart disease by reducing the risk of new cardiovascular events. Among these effects, statins are able to modulate the host immune response [9]. This probably results from a wide range of interferences with proinflammatory pathways. Using statin therapy to protect patients with sepsis could therefore be a reasonable strategy, as these drugs could dampen an overwhelming host inflammatory response to infection, especially within the lung [28]. Many studies, mainly observational, have addressed this issue [15]. However, contrasting findings have been reported. This may result from both study design and the widely varying characteristics of the populations included. All in all, the protective effect of statins during sepsis remains an unsolved issue.

Ventilator-associated pneumonia remains one of the major complications of protracted stays in the ICU. Despite early and appropriate antibiotics, the response rates to antibacterial therapy remain low. This could result from deep alterations of the host immune response in this setting, as a shift toward a proinflammatory state occurs within the lung if subjected to MV. This proinflammatory state is likely to weaken the host’s ability to clear pathogens [29]. As suggested in previous studies [20], statin therapy may therefore have a beneficial effect in patients with VAP.

We report herein the findings from a single-center cohort study that investigated, retrospectively, the effects of statin therapy on the outcome of patients with suspected VAP. In the first set of analyses, we compared statin-naive patients with those who received statins, whether or not treatment was discontinued upon admission to the ICU. We found that mortality was not different between the groups, and this despite the fact that previous users were older and sicker. A multivariate survival analysis was then conducted but failed to demonstrate any difference in survival despite multiple adjustments. Moreover, various relevant variables may have been missing from our analysis, especially because of the retrospective design of our study. Thus, additional atherosclerosis risk factors such as smoking or obesity, which are frequently associated with statin intake, could act as masking confounders. Unfortunately, we were unable to adjust for such factors, thus making it more difficult to demonstrate any beneficial effect.

We therefore conducted a second set of analyses restricted to patients with prior statin therapy, in an attempt to overcome this issue. This approach was also supported by the fact that it remains unknown whether statins should be continued or not when patients on statin therapy present with a critical illness requiring treatment in an ICU, notably because of toxicity concerns. Similar to our first findings, we found no statistically significant difference between patients who continued statin therapy and those who did not after admission to the ICU. However, after adjusting for potential confounders, continued statin therapy was found to be independently related to survival in the ICU. Several explanations could be provided. First, this subset of patients (that is, previous statin users) was probably more homogeneous regarding cardiovascular risk factors and associated illnesses, thereby making the existence of uncontrolled confounding variables less likely. Second, the protective effect of statins during sepsis may decrease with the duration of discontinuation. Obviously, the most consistent published data were obtained in patients hospitalized with severe community-acquired pneumonia, that is to say patients actually exposed to statins at least until the onset of sepsis [13,14]. If statins were discontinued on admission, it is likely that the anti-inflammatory effects of statins were diminished if not abolished when the VAP occurred, that is to say several days, if not weeks, later. This is especially true in our cohort since most of the VAP episodes recorded were late-onset. Conversely, a previously published RCT showed that in previous statin users hospitalized with signs of infection, drug continuation was not associated with a better outcome [30]. It is worth noting that illness severity was mild in this study. Indeed, the same authors have reported more recently the results of another RCT comparing atorvastatin 20 mg daily to placebo given upon ICU admission to critically ill patients with sepsis [21]. These authors showed that in the predefined subgroup of patients who received statins prior to ICU admission there was a significant decrease in 28-day mortality in the treatment arm when compared to placebo, whereas no effect was measured if the new users (that is, statin-naive patients) were also considered. It is worth noting that in our cohort, we did not find any statin new users. We cannot therefore conclude about the putative protective effect of statin *de novo* treatment in the ICU, an issue addressed by several ongoing or recently completed RCTs. Among them, the ‘STATIN VAP’ study showed that no beneficial effect could be expected from simvastatin when given in patients with suspected VAP [22]. Although speculative, one can hypothesize that statin anti-inflammatory effects, if any, could be obtained and protect the host as well only if given prior to infection.

Several limitations should, however, be mentioned. First, although most of the data were collected prospectively, information regarding statin use was acquired retrospectively and could therefore be a matter of concern. However, we systematically excluded from the analysis patients with unreliable or missing data. Second, because of the retrospective design of the study, the risk of missing confounding data is real. Our findings should thus be interpreted with caution. Third, the assessment of renal function was based on serum creatinine alone, which is subject to criticism, especially in the ICU setting. Fourth, liver test abnormalities on admission were not recorded in our database. We cannot exclude that statin discontinuation was thus influenced in some patients, accounting thereby for differences of outcome. In addition, we were unable to demonstrate the direct detrimental effects of statin in our patients since, for instance, creatine kinase levels were not available. Some of our conclusions are therefore only speculative. Moreover, we did not record which type of statin was used in the included patients, despite the fact that anti-inflammatory properties may differ according to the considered subclass (that is, lipophilic vs. hydrophilic). Similarly, patients were classified as previous users if any statin therapy has been given prior to ICU admission regardless of treatment duration, making unlikely any conclusion about the minimal required time of exposure. In addition, inflammation assessment relied on the sole PCT measurement. Inflammatory cytokines assessment would have maybe provided different insights regarding host response. However, PCT monitoring has been shown to be clinically relevant in patients with VAP [31]. Moreover, we cannot exclude that some patients without confirmed VAP were included in the study since we used diagnosis criteria known to lack of specificity [24]. As a result, any possible protective effect of statins in our cohort may be independent from VAP occurrence. However, since we considered the only episodes in which the physician in charge promptly delivered antibiotics, we believe that it reflects real-life practice. In addition, the CPIS value on day 3 was rather high (6.5 (2) points within the whole cohort), strongly suggesting thereby VAP diagnosis in our patients. Moreover, it is worth noting that such a beneficial effect on survival of statin exposure was also noticed in the subset of patients of microbiologically proven VAP. Finally, the sample size in our subgroup analysis was quite small, raising the risk of a type-2 error because of a lack of statistical power.

## Conclusions

In our cohort, continuing statins after admission to an ICU may have a protective effect in patients with suspected VAP.

## Key messages

• Statins protective effects in patients with pneumonia as well as drug continuation after ICU admission are unsolved issues.

• In our cohort of patients with suspected ventilator-associated pneumonia, statin continuation in previous users was shown to be an independent predictor of survival.

## Abbreviations

CFU: colony-forming unit; CI: confidence interval; CPIS: clinical pulmonary infection score; HR: hazard ratio; ICU: intensive care unit; MDR: multidrug resistant; MV: mechanical ventilation; PCT: procalcitonin; RCT: randomized controlled trial; SAPS II: simplified acute physiology score II; SOFA: sequential organ failure assessment; VAP: ventilator-associated pneumonia; VILI: ventilator-induced lung injury.

## Competing interests

The authors declare they have no competing interests.

## Authors’ contribution

PEC, RB and LP designed the study. PEC, RB, CV, SP and JPQ collected the data. AP collected the microbiological data and analyzed them. PEC drafted the manuscript. PEC, RB and SA performed statistical analysis of the data. RB, CV, SP, SA, AP, LP and JPQ critically revised the manuscript. All the authors approved the final version to be published.
